# Diagnostic accuracy of ultra-low-dose CT for torsion measurement of the lower limb

**DOI:** 10.1007/s00330-020-07528-8

**Published:** 2020-11-25

**Authors:** Gabriel Keller, Saif Afat, Marc-Daniel Ahrend, Fabian Springer

**Affiliations:** 1 Department of Diagnostic and Interventional Radiology, University Hospital Tübingen, Eberhard Karls University Tübingen, Hoppe-Seyler-Str. 3, 72076 Tübingen, Germany; 2 Department of Diagnostic Radiology, BG Trauma Center Tübingen, Eberhard Karls University Tübingen, Schnarrenbergstr. 95, 72076 Tübingen, Germany; 3 Department of Traumatology and Reconstructive Surgery, BG Trauma Center Tübingen, Eberhard Karls University of Tübingen, Schnarrenberg-Str. 95, 72076 Tübingen, Germany

**Keywords:** Radiation exposure, Radiation dosage, Rotation, Lower limb, Lower extremity

## Abstract

**Objectives:**

The study aimed to investigate the diagnostic performance of simulated ultra-low-dose CT (ULD-CT) for torsion measurement of the lower limb.

**Methods:**

Thirty retrospectively identified patients were included (32.3 ± 14.2 years; 14 women, 16 men). ULD-CT simulations were generated at dose levels of 100%, 10%, 5%, and 1% using two reconstruction methods: standard filtered back projection (FBP) and iterative reconstruction (ADMIRE). Two readers measured the lower limb torsion in all data sets. The readers also captured image noise in standardized anatomical landmarks. All data sets were evaluated regarding subjective diagnostic confidence (DC; 5-point Likert scale). Effective radiation dose of the original data sets and the simulated ULD-CT was compared.

**Results:**

There was no significant difference of measured lower limb torsion in any simulated dose level compared to the original data sets in both readers. Dose length product (DLP) of the original examinations was 402.1 ± 4.3 mGy cm, which resulted in an effective radiation dose of 4.00 ± 2.12 mSv. Calculated effective radiation dose in ULD-CT at 1% of the original dose was 0.04 mSv. Image noise increased significantly with dose reduction (*p* < 0.0001) and was dependent on the reconstructional method (*p* < 0.0001) with less noise using ADMIRE compared to FBP. Both readers rated DC at doses 100%, 10%, and 5% with 5.0/5: there were no ratings worse than 3/5 at 1% dose level.

**Conclusions:**

The results suggest that radiation dose reduction down to 1% of original CT dose levels may be achieved in CT torsion measurements of the lower limb without compromising diagnostic accuracy.

**Key Points:**

*• Modern CT delivers exceptional high image quality in musculoskeletal imaging, especially for evaluation of osseous structures.*

*• Usually, this high image quality is accompanied by significant radiation exposure to the patient and may not always be required for the intended purpose, e.g., pure delineation of cortical bone of the lower limb.*

*• This study shows the tremendous prospects of radiation dose reduction without compromising diagnostic confidence in CT torsion measurement of the lower limb.*

## Introduction

Torsional malalignment of the lower limb is a common clinical problem in adults and children. It is a predisposing factor for anterior knee pain [1] and has a significant impact on gait as well as daily-life activity [2]. Congenital variant torsional malalignment predisposes for recurrent patellar dislocations especially in adolescents but may also occur in a post-traumatic setting following fracture of the femur or tibia. Thus, torsion measurement of the lower limb plays a significant role in the treatment and pre-operative planning of several non-traumatic and traumatic conditions of the lower extremity [2]. The German Society for Trauma Surgery explicitly recommends torsion measurement of the lower extremity as part of a thorough clinical workup after recurrent patellar dislocations [3–5]. Since multiple studies have shown that torsional malalignment of the lower extremity correlates with the development of osteoarthritis, operative correction of lower limb torsion may prevent deterioration or development of osteoarthritis in the long term [6–8].

To date, computed tomography (CT) is considered the gold standard for the clinical workup of lower extremity malalignment [9–11]. Non-contrast-enhanced CT is widely available, fast, and cost-effective compared to other methods such as magnetic resonance imaging (MRI). Nevertheless, CT imaging comes at the cost of significant radiation exposure which limits its applicability especially in the most radiation-sensitive part of the population such as children and young adults. Cumulative radiation dose—caused by multiple CT scans used for pre- and post-operative torsion measurement of the lower limb—may increase their cancer risk. Therefore, it is crucial to optimize CT acquisition protocols and use the fundamental principle of radiation protection, the “as low as reasonably achievable (ALARA)” principle, without compromising the diagnostic accuracy of torsion measurements.

Various approaches have been evaluated to reduce the radiation dose using either new devices or alternative modalities [9, 12–15]. Most recently, Yan et al published an article comparing torsion measurement between a whole-body low-dose X-ray system and a standard CT with the main focus on the agreement between the two systems and not the reduction of the radiation dose of CT scans [15]. However, there have already been different approaches investigated to reduce radiation dose generally in CT imaging by modifying either the scan parameters or by using dedicated post-processing software [16, 17]. But to our knowledge, among articles on the diagnostic workup of torsional malalignment of the lower limb, there are no studies published evaluating the maximum possible dose reduction of CT scans for torsion measurement of the lower limb. In this retrospective study, we aimed to compare the diagnostic performance, image quality, and estimated effective radiation dose of a simulated ultra-low-dose CT (ULD-CT) using an intra-individual comparison to the original data set of patients who underwent a clinically indicated CT scan for torsion measurements of the lower limb. Furthermore, we also analyzed the influence of two different post-processing methods by using advanced modeled iterative CT image reconstruction and compared it to regular filtered back projection CT image reconstruction.

## Methods

### Study population

Thirty patients (14 female, 16 male; mean age of 32.3 ± 14.2 years; range 15–62 years) were randomly selected from all patients who received a clinically indicated CT torsion measurement of the lower limb in 2018 and for which the raw data files were still available. Exclusion criteria were metal implants, e.g., joint arthroplasty of the hip, knee, or ankle joint. The study protocol was approved by the institutional review board (review board application number 626/2019BO2) and the need for written informed consent was waived for this retrospective analysis of clinically acquired data.

### Technical parameters of the original CT

Original CT image acquisition was performed using a single-source CT (SOMATOM Definition Edge, Siemens Healthineers) using a standard study protocol based on automated tube current modulation for individual patient size and shape using CARE Dose4D (Siemens Healthineers). Tube voltage was set to 120 kV and different reference tube currents for the hip (220 mAs), knee (95 mAs), and ankle (95 mAs) regions were set. Pitch was 1.0, rotation time 0.5 s, and collimation 128 × 0.6 mm.

### ULD-CT simulation

**Fig. 1:**
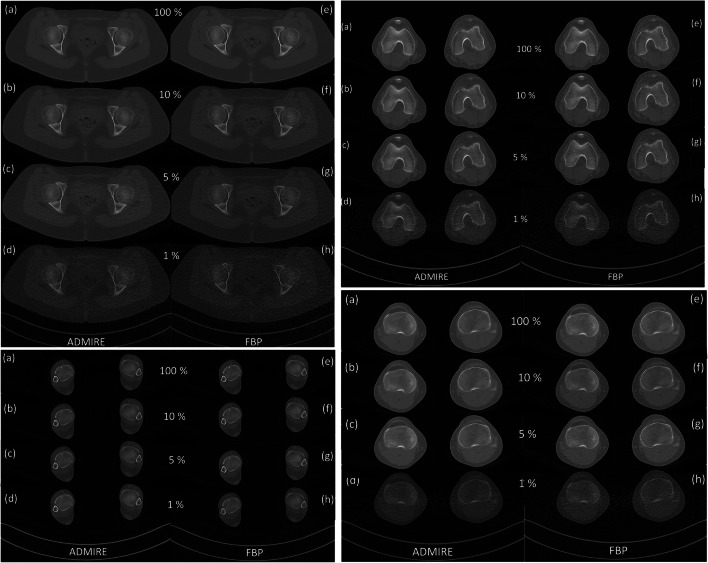
A 17-year-old patient who received a preoperative CT torsion measurement of the lower limb; separately shown are the hip, knee, and ankle regions. Simulated ULD-CT data sets are shown with radiation dose of 10% (b), 5% (c), and 1% (d) of the original dose as well as the original CT data set with 100% radiation dose (a). Iterative reconstruction (ADMIRE) was used in the left column; filtered back projection (FBP) in the right column (e-h)

Using raw data from CT torsion measurements of the lower extremity, ULD-CT simulations were generated with the dedicated software package ReconCT (Version 14.2.0.40998, Siemens Healthineers) [18, 19]. Simulations were made at 10%, 5%, and 1% dose levels and compared to the original data sets (100% dose level). The software ReconCT essentially generates images with reduced radiation dose by adding noise to the raw data prior to the reconstruction process. Axial reformats (3-mm slice thickness; sharp edge–enhancing reconstruction Kernel B60; window center 450/width 1500) at every dose level were made twice using two different methods of raw data reconstruction: standard filtered back projection (FBP) and advanced modeled iterative reconstruction (ADMIRE Stage 5, Siemens Healthineers) (Fig. 1). Iterative reconstruction was used at the highest possible stage (stage 5) in order to achieve maximum possible noise reduction at the cost of slightly increased image blurring and reduced visibility of detail (but not necessary for delineation of cortical outline of bone).

### Estimation of the effective radiation dose

For calculation of radiation dose, a dedicated software package was used (Radimetrics Enterprise Platform, Bayer Pharma). Within this software package, the X-ray source spectrum is based on the model described in the National Radiological Protection Board (NRPB) R204.

The effective dose is defined as the sum of equivalent doses from all organs, weighted by tissue weighting factors from the International Commission on Radiological Protection (ICRP103) and is directly calculated using Monte Carlo simulations. Tube current modulation in the *z*-axis (CareDose 4D, Siemens Healthineers) was taken care of. No gantry tilt or tube current modulation in the *x*-*y* plane was used in both CT protocols, and thus did not have to be accounted for.

### Torsion measurements

**Fig. 2:**
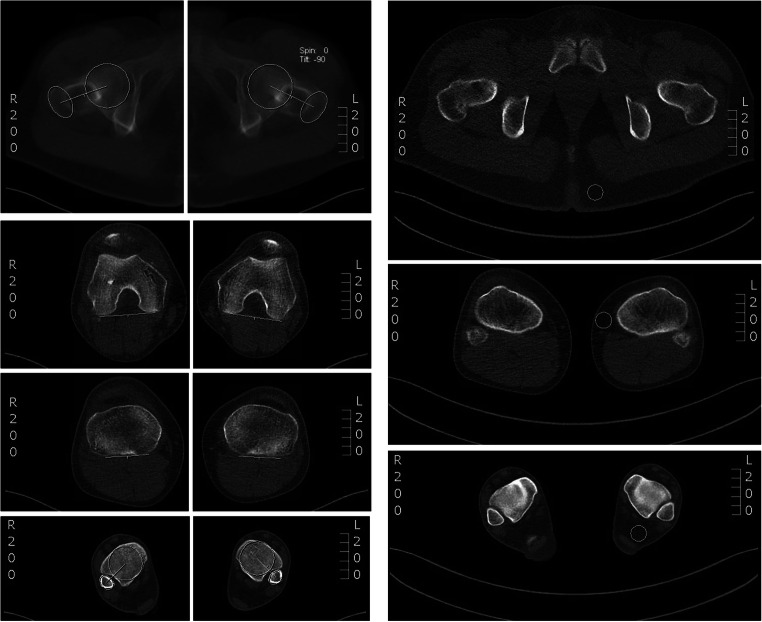
CT torsion measurement of the lower limb using a technique modified from Waidelich et al [20] and measurement of image noise in fatty tissue at standardized anatomical landmarks

Two readers (G.K. and S.A.) with 3 and 5 years of experience in musculoskeletal imaging measured the lower limb torsion in all data sets using a technique modified from Waidelich et al [20]: Femoral torsion was measured as the angle between a line central through the femoral head and central through the greater trochanter (femoral neck) and a second line along the posterior margin of the femoral condyles. Tibial torsion was measured between a line along the posterior margin of the tibial plateau and a line central through the tibial and fibular parts of the ankle joint. Overall torsion of the lower limb was measured between the femoral neck and ankle joint (Fig. 2).

As an objective measure, the image noise in fatty tissue was captured within a 2-cm^2^ circular region of interest (ROI) tool at standardized anatomical landmarks next to the rima ani (hip scan), next to the medial tibia plateau (knee scan), and next to the Achilles’ tendon (ankle scan) (Fig. 2). In addition, both readers (G.K. and S.A.) rated their subjective diagnostic confidence level regarding the identification of the relevant cortical bone in every simulated data set as well as in the original data set on a 5-point Likert scale from “poor” (1) to “excellent” (5). The hip, knee, and ankle scans were rated independently.

### Statistical analysis

Statistical analysis was made using JMP (Version 14.2.0, SAS Institute Inc.) and SPSS (Version 25.0.0.1, IBM Corp.). Arithmetic mean and standard deviation were calculated for femoral, tibial, and overall torsion of the lower limb, dose length product (DLP), and image noise. Continuous variables such as DLP, effective radiation dose, image noise, and torsion were checked for normality by the Shapiro-Wilk *W* test. Normally distributed variables were analyzed using the Student *t* test, and non-normally distributed variables using the Wilcoxon test. Correlations of ordinal variables such as the individual subjective diagnostic confidence (DC) were analyzed by likelihood ratio. Variables were compared between different dose levels and methods of reconstruction. *p* values < 0.05 indicate statistical significance. Furthermore, torsion measurements at all reduced dose levels were compared to those measured at 100% radiation dose using Bland-Altman plots and evaluated regarding any systematic error or proportional bias as well as the level of agreement. Inter-reader agreement was calculated using intra-class correlation coefficients (ICC).

## Results

Mean overall DLP of CT torsion measurements of the lower limb was 402.1 ± 84.3 mGy cm for the original data sets. With a simulated dose reduction, overall DLP values decreased to 4.0 ± 0.8 mGy cm at 1% of the original dose (Table 1). Total effective radiation dose of the original CT torsion measurement data sets was 4.00 ± 2.12 mSv. Subdivided into the three parts of the examination, effective radiation doses were 3.46 ± 1.75 mSv at the hip level, 0.51 ± 0.68 mSv at the knee level, and 0.02 ± 0.02 mSv at the ankle level. Total effective radiation dose estimation significantly decreased to 0.04 ± 0.02 mSv at 1% (Table 2).

**Table 1:** Image noise, DLP, and DC at different dose levels

Dose	Reco	Hip			Knee			Ankle			DLP (mGy cm)
		Noise (HU)	DC 1	DC 2	Noise	DC 1	DC 2	Noise	DC 1	DC 2	
100%	AD	33.9 ± 11.2	5.0/5	5.0/5	31.5 ± 12.5	5.0/5	5.0/5	23.9 ± 11.4	5.0/5	5.0/5	402.1 ± 84.3
	FBP	55.5 ± 16.2	5.0/5	5.0/5	49.8 ± 18.5	5.0/5	5.0/5	41.4 ± 16.3	5.0/5	5.0/5	402.1 ± 84.3
10%	AD	100.8 ± 23.4	5.0/5	5.0/5	71.3 ± 15.6	5.0/5	5.0/5	43.9 ± 12.2	5.0/5	5.0/5	40.2 ± 8.4
	FBP	166.8 ± 39.4	5.0/5	5.0/5	123.6 ± 24.0	5.0/5	5.0/5	72.5 ± 15.2	5.0/5	5.0/5	40.2 ± 8.4
5%	AD	154.6 ± 38.3	5.0/5	5.0/5	111.5 ± 34.4	5.0/5	5.0/5	59.7 ± 16.8	5.0/5	5.0/5	20.1 ± 4.2
	FBP	260.5 ± 77.3	5.0/5	5.0/5	176.7 ± 32.4	5.0/5	5.0/5	99.1 ± 23.3	5.0/5	5.0/5	20.1 ± 4.2
1%	AD	754.4 ± 178.0	4.5/5 ± 0.5	4.4/5 ± 0.6	347.7 ± 89.6	4.8/5 ± 0.3	4.7/5 ± 0.4	135.7 ± 56.1	5.0/5	5.0/5	4.0 ± 0.8
	FBP	865.2 ± 150.8	4.1/5 ± 0.6	4.1/5 ± 0.7	563.6 ± 103.8	4.6/5 ± 0.5	4.4/5 ± 0.6	231.5 ± 77.5	4.9/5 ± 0.1	4.9/5 ± 0.2	4.0 ± 0.8

Radiation dose of original data (100%) of CT torsion measurements of the lower limb (*n* = 30 patients) and calculated radiation dose of simulated ULD-CT at dose levels 10%, 5%, and 1% using iterative reconstruction and standard filtered back projection. Image noise and subjectively rated diagnostic confidence regarding the identification of the relevant cortical bone for the torsion measurement on a 5-point Likert scale (1 = poor, 5 = excellent) of two readers in original data sets (100%) subdivided into hip, knee, and ankle scans of CT

*Reco* method of reconstruction, *DC* subjective diagnostic confidence, *DLP* dose length product, *Noise* image noise, *AD* iterative reconstruction, *FBP* standard filtered back projection

**Table 2:** Effective radiation dose at different dose levels

Dose	Hip (mSv)	Knee (mSv)	Ankle (mSv)	Total (mSv)
Original	3.46 ± 1.75	0.51 ± 0.68	0.02 ± 0.02	4.00 ± 2.12
10%	0.35 ± 0.18	0.05 ± 0.07	< 0.01	0.40 ± 0.21
5%	0.17 ± 0.09	0.03 ± 0.03	< 0.01	0.20 ± 0.11
1%	0.03 ± 0.02	< 0.01	< 0.01	0.04 ± 0.02

Effective radiation dose of original data (100%) and at simulated ULD-CT dose levels of CT torsion measurements of the lower limb (*n* = 30 patients) subdivided into hip, knee, and ankle scans and total effective radiation dose of the CT

In the original data sets at a 100% dose level, mean image noise was highest at the hip level and lowest at the ankle level (Table 1). Image noise increased significantly with a lower effective radiation dose level (*p* < 0.0001). It was also significantly lower in reconstructions using iterative methods (ADMIRE) compared to standard filtered back projection (FBP) (*p* < 0.0001) (Table 1).

**Fig. 3:**
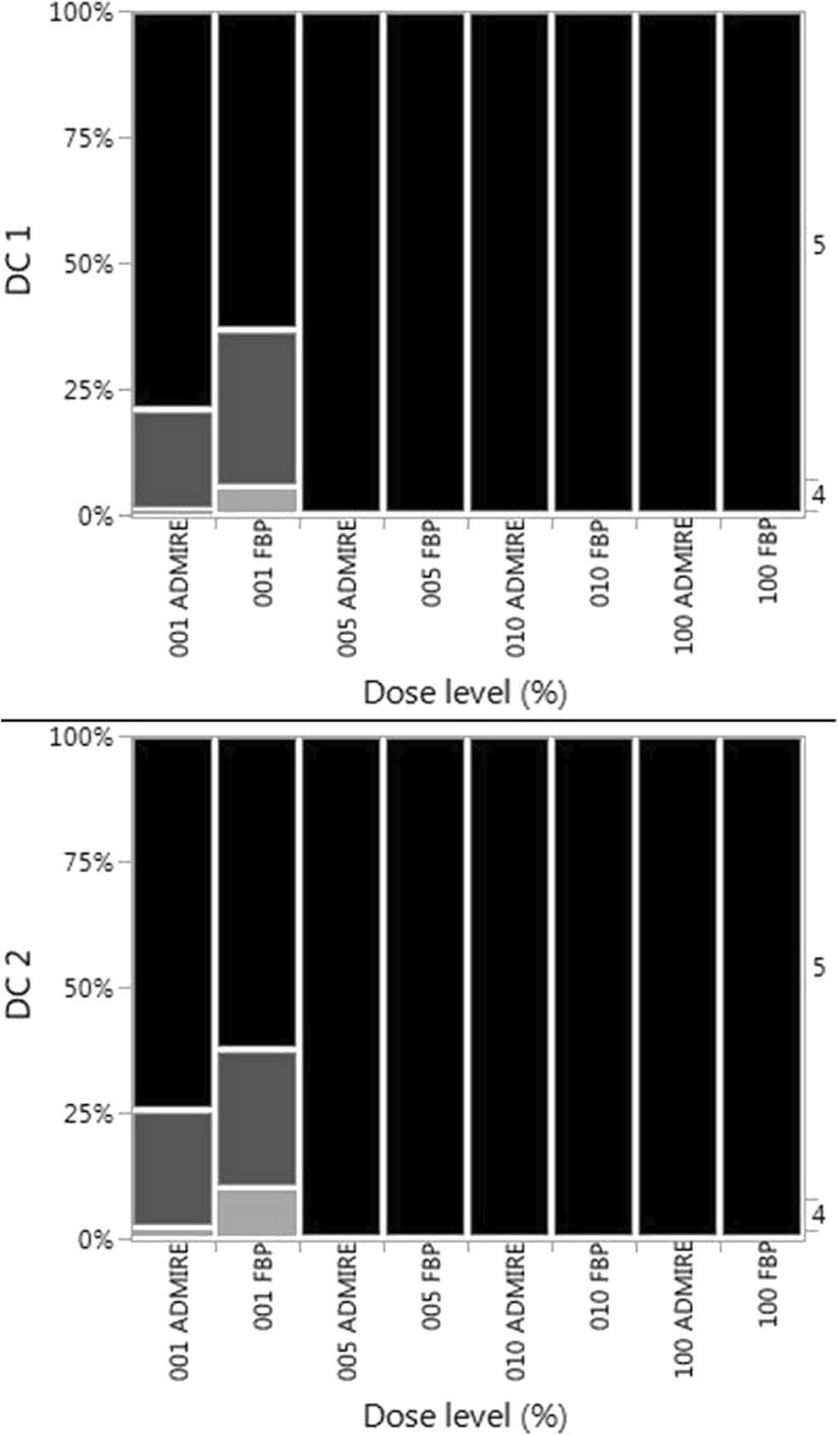
Subjectively rated diagnostic confidence (DC) shown for reader 1 (DC 1) and for reader 2 (DC 2) for different dose levels and methods of reconstruction (e.g., 001 ADMIRE = 1% simulated dose iterative reconstructed; 010 FBP = 10% simulated dose reconstructed using standard filtered back projection). Black represents an excellent rating (5/5 on the 5-point Likert scale), darker gray 4/5, and lighter gray 3/5. There was no rating lower than 3/5

Both readers rated the diagnostic confidence (DC) in the original data sets and in every simulated CT reconstruction at 100%, 10%, and 5% dose levels with 5/5. The worst rated subgroup were the hip scans at 1% dose level reconstructed with FBP, demonstrating only 23.3% (reader 1) and 36.7% (reader 2) excellent ratings (5/5), 63.3%/43.3% ratings of 4/5 for readers 1 and 2, and 13.3%/20.0% ratings of 3/5 for readers 1 and 2 on a 5-point Likert scale. There was no rating lower than 3/5 in any simulated CT data set. For both readers, a lower simulated dose level correlated significantly with a lower DC (*p* < 0.0001) (Table 1; Fig. 3).

**Fig. 4:**
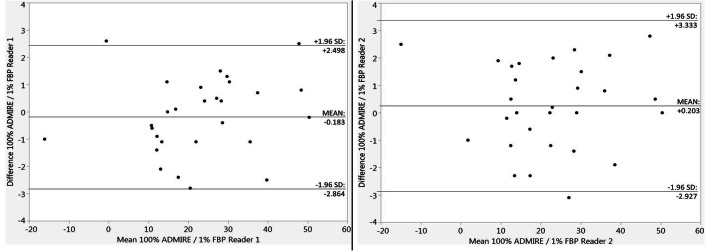
Exemplary Bland-Altman plots for measurement of the overall torsion of the lower limb in original data sets and in the ULD-CT simulations at 1% of the original dose reconstructed with FBP—the simulated ULD-CT with the most noise and the worst rated DC, shown separately for reader 1 (left) and reader 2 (right). FBP standard filtered back projection, DC subjective diagnostic confidence

Overall mean torsion of the lower limb in the original data sets was measured to be 22.6° ± 14.3° (reader 1) and 23.0° ± 14.0° (reader 2) with no significant differences for all investigated dose levels and methods of reconstruction (Table 3; Fig. 4). There were no systematic errors or proportional biases between the original data and simulated low-dose levels as evaluated by Bland-Altman plots. Mean difference of the two readers in the overall torsion measurement of the lower limb was 0.1° ± 1.3. This resulted in an ICC for torsion measurement of 0.99 (95% CI, 0.997–0.998). The ICC for femoral torsion was 0.99 (95% CI, 0.995–0.997); the ICC for tibial torsion was 0.99 (95% CI, 0.991–0.994).

**Table 3:** Torsion measurements at different dose levels

Reco	Dose (%)	Torsion 1 (°)	*p* value	Torsion 2 (°)	*p* value	Femoral 1 (°)	Femoral 2 (°)	Tibial 1 (°)	Tibial 2 (°)
AD	100	22.6 ± 14.5		23.0 ± 14.2		20.4 ± 10.7	20.3 ± 10.4	40.1 ± 11.1	40.3 ± 11.1
	10	22.5 ± 14.4	0.88	22.4 ± 14.3	0.97	20.4 ± 10.7	20.5 ± 10.6	40.2 ± 11.4	40.5 ± 11.4
	5	22.6 ± 14.0	1.00	22.6 ± 13.9	0.95	20.7 ± 10.8	20.5 ± 10.5	40.5 ± 11.2	40.3 ± 11.1
	1	22.3 ± 14.9	0.91	22.6 ± 14.3	0.94	20.7 ± 11.2	20.2 ± 10.8	40.5 ± 11.1	40.1 ± 11.1
FBP	100	22.8 ± 14.1	0.92	22.9 ± 14.2	1.00	20.6 ± 10.8	20.5 ± 11.1	40.7 ± 11.0	40.5 ± 11.1
	10	22.8 ± 14.2	0.95	22.7 ± 14.5	0.85	20.6 ± 10.8	20.7 ± 11.3	40.6 ± 11.1	40.1 ± 11.4
	5	22.7 ± 14.0	0.87	22.7 ± 14.5	0.87	20.6 ± 10.9	20.5 ± 11.2	40.3 ± 11.2	40.1 ± 11.1
	1	22.8 ± 14.2	0.92	22.7 ± 14.1	0.87	20.6 ± 11.3	20.7 ± 11.0	40.5 ± 11.0	40.3 ± 10.9

Overall external torsion of the lower limb, femoral torsion, and tibial torsion measured by two readers in original CT data sets (100%) and in simulated ULD-CT (10% dose, 5% dose, 1% dose) using iterative reconstruction and standard filtered back projection

*Reco* method of reconstruction, *Dose* radiation dose, *Torsion 1/2* measured overall torsion of the lower limb of reader 1/2, *Femoral 1/2* measured femoral torsion of reader 1/2, *Tibial 1/2* measured tibial torsion of reader 1/2, *AD* iterative reconstruction, *FBP* standard filtered back projection

## Discussion

Radiation dose and different strategies for dose reduction especially in CT imaging have been a hot topic in the medical community for many years. In the presented study, we have set the focus on modifying CT acquisition parameters for torsion measurement of the lower limb which is easy to accomplish, does not necessarily require additional hardware or sophisticated post-processing software, and can be performed on various CT scanner generations. By doing so, we aimed to explore the boundaries of the ALARA principle by simulating ULD-CT images.

We evaluated the diagnostic performance of ULD-CT for torsion measurement of the lower limb in 30 patients. The diagnostic accuracy of ULD-CT at all dose levels showed no significant differences compared to the original data set. Although image noise measured in a homogenous area of subcutaneous fat increased significantly with the reduction of radiation dose, there was no significant difference in measured torsion at all simulated dose levels compared with the torsion measurement of the original data set. Additionally, we were able to show high levels of inter-reader agreement in terms of diagnostic accuracy. These findings indicate that ULD-CT is suitable for measuring torsional alignment of the lower limb in a clinical setting.

To date, many studies have shown that ULD-CT can be a useful alternative to conventional radiography for imaging of extremities, e.g., in a post-traumatic setting [21–23]. These results have recently been verified by Konda et al showing a high sensitivity (98%) of ULD-CT in detecting fractures [24]. Also, Yi et al reported that low-dose CT of the shoulder, pelvis, ankle, and wrist could be reduced by 50% with no significant difference in diagnostic performance compared to standard-dose CT [25]. These findings are consistent with the results of our study. If fractures can be confidently depicted on low-dose CT scans, the cortical outline of a bone may be easily delineated even on ultra-low-dose CT images.

Apart from reduction of effective tube current and advanced post-processing algorithms such as iterative image reconstruction, another effective method to reduce radiation dose is to reduce the tube current. Low kilovolt protocols using 100 kV or 80 kV might even allow further reduction of radiation dose, but depending on body size they may not all be suitable for the hip region or for a post-operative setting when internal or external fixation material is present. However, low kilovolt protocols have already been used in fracture detection [24, 25]. Automatic tube voltage adjustment (e.g., CARE kV, Siemens Healthineers) may partly overcome this problem but is not widely available especially on older scanners [26, 27]. Furthermore, the use of tin filters for spectral shaping may additionally reduce radiation dose, and these have been used in various settings, but are also not available on all scanners [28, 29].

Apart from the abovementioned technical parameters, the easiest way to reduce effective radiation dose is to reduce the scan range at each level to the shortest length necessary for accurate torsion measurement. This potential for dose reduction is greatest for the hip region, and thus scan length should only include the greater trochanter and femoral head. Use of an adaptive collimation may additionally decrease radiation dose [30, 31].

It is known that the lower limbs have relatively low radiation sensitivity, but measurement of lower limb torsion always includes parts of the pelvis when scanning the hip region comprising radiation-sensitive organs such as the testicles or ovaries. For example, the mean effective dose for a conventional radiograph of the pelvis amounts to about 0.6 mSv (calculated at www.xrayrisk.com), whereas in our study the whole ULD-CT at the 1% dose level including the hip, the knee, and the ankle results in an effective radiation dose of only 0.04 mSv. This finding demonstrates that a significant radiation dose reduction for torsion measurement is achievable even on conventional CT scanners. Konda et al also recently reported an estimated effective dose of only 0.24 mSv in the hip region by using an ULD-CT protocol for detecting fractures. While our study is serving another purpose, the effective dose estimation of 0.24 mSv is still much higher compared to our 1% dose level approach with only 0.03 mSv in the hip region [24].

In contrast to our approach, previously published studies regarding radiation dose reduction in torsion measurement of the lower limb mostly focused on using other modalities [12–14]. For example, Meyrignac et al showed in a prospective setting that using a dedicated biplanar device torsion measurement of the lower extremity is comparable to CT scans with a lower radiation dose. However, this approach is not widely applicable due to the need to purchase new devices and may even be deemed superfluous in view of our findings regarding the achievable radiation dose reduction in ultra-low-dose CT.

Our study has some limitations. First, the retrospective design of the study from a single center can be regarded as a limitation. We have used CT scans from the clinical routine to evaluate possibilities of dose reduction retrospectively in order to prevent false torsional measurements of the lower limb with possible clinical consequences. However, the ultra-low-dose simulation of CT data sets is a proven and accurate method to evaluate low-dose CT images but is nevertheless a simulation. Therefore, further in vivo studies are warranted to demonstrate the ULD-CT approach for torsion measurements in vivo. The simulated data sets can only add noise to the raw data in order to achieve low-dose images: detailed possibilities such as adjustment of effective tube current, lower tube voltage settings, or tin filtration cannot be simulated adequately. By using low kilovolt protocols comprising 100-kV or even 80-kV tube voltages, a diagnostic image quality may be achieved at even lower radiation exposure. However, since we excluded patients with metal implants, we can not conclude about CT torsion measurements in patients after internal fixation with intramedullary nailing or arthroplasties.

In summary, our study demonstrates that ULD-CT is a significant advance in torsion measurement of the lower limb and results in significant radiation dose reduction without compromising diagnostic confidence in the clinical workup.

## Abbreviations

ADMIRE: Advanced modeled iterative reconstruction (Siemens Healthineers)
ALARA: “As low as reasonably achievable” principle
DC: Subjective rated diagnostic confidence
DLP: Dose length product
FBP: Filtered back projection
ICC: Intra-class correlation coefficient
ROI: Region of interest
ULD-CT: Ultra-low-dose computed tomography
