# Correction to: Diagnostic accuracy of ultra-low-dose CT for torsion measurement of the lower limb

**DOI:** 10.1007/s00330-021-07691-6

**Published:** 2021-01-25

**Authors:** Gabriel Keller, Saif Afat, Marc-Daniel Ahrend, Fabian Springer

**Affiliations:** 1 Department of Diagnostic and Interventional Radiology, University Hospital Tübingen, Eberhard Karls University Tübingen, Hoppe-Seyler-Str. 3, 72076 Tübingen, Germany; 2 Department of Diagnostic Radiology, BG Trauma Center Tübingen, Eberhard Karls University Tübingen, Schnarrenbergstr. 95, 72076 Tübingen, Germany; 3 Department of Traumatology and Reconstructive Surgery, BG Trauma Center Tübingen, Eberhard Karls University of Tübingen, Schnarrenberg-Str. 95, 72076 Tübingen, Germany

**Correction to: European Radiology**

10.1007/s00330-020-07528-8

The original version of this article, published on 25 November 2020, unfortunately contained a mistake. The following correction has therefore been made in the original: The information that Gabriel Keller and Saif Afat share the first authorship was missing. The original article has been corrected.

The article “Diagnostic accuracy of ultra-low-dose CT for torsion measurement of the lower limb”, written by Keller, G., Afat, S., Ahrend, MD. and Springer, F., was originally published Online First without Open Access. After publication in volume 31, issue 6, page 3574–3581 the author decided to opt for Open Choice and to make the article an Open Access publication. Therefore, the copyright of the article has been changed to © The Author(s) 2020 and the article is forthwith distributed under the terms of the Creative Commons Attribution 4.0 International License, which permits use, sharing, adaptation, distribution and reproduction in any medium or format, as long as you give appropriate credit to the original author(s) and the source, provide a link to the Creative Commons licence, and indicate if changes were made.

The images or other third party material in this article are included in the article’s Creative Commons licence, unless indicated otherwise in a credit line to the material. If material is not included in the article’s Creative Commons licence and your intended use is not permitted by statutory regulation or exceeds the permitted use, you will need to obtain permission directly from the copyright holder.

To view a copy of this licence, visit http://creativecommons.org/licenses/by/4.0/.

